# Network pharmacology -based study on the mechanism of traditional Chinese medicine in the treatment of glioblastoma multiforme

**DOI:** 10.1186/s12906-023-04174-7

**Published:** 2023-09-27

**Authors:** Chen Liang, Binbin Zhang, Ruichun Li, Shiwen Guo, Xiaoxuan Fan

**Affiliations:** 1 Department of Neurosurgery, The First Affiliated Hospital of Xi’an Jiaotong University, Xi’an, 710061 China; 2 Division of Medical Physics, Department of Diagnostic and Interventional Radiology, University Medical Center Freiburg, Faculty of Medicine, University of Freiburg, 79108 Freiburg, Germany; 3 Affiliated Hospital of Shaanxi University of Chinese Medicine, Xianyang, 712000 China

**Keywords:** Network pharmacology, Traditional Chinese medicine, Glioblastoma multiforme, Molecular docking

## Abstract

**Background:**

Glioblastoma multiforme (GBM) is one of the most common primary malignant brain tumors. Yi Qi Qu Yu Jie Du Fang (YYQQJDF) is a traditional Chinese medicine (TCM) prescription for GBM. The present study aimed to use a network pharmacology method to analyze the underlying mechanism of YQQYJDF in treating GBM.

**Methods:**

GBM sample data, active ingredients and potential targets of YQQYJDF were obtained from databases. R language was used to screen differentially expressed genes (DEGs) between GBM tissues and normal tissues, and to perform enrichment analysis and weighted gene coexpression network analysis (WGCNA). The Search Tool for the Retrieval of Interacting Genes/Proteins (STRING) database was used to perform a protein‒protein interaction (PPI) analysis. A Venn diagram was used to obtain the core target genes of YQQYJDF for GBM treatment. Molecular docking was used to verify the binding between the active ingredient molecules and the proteins corresponding to the core target genes. Cell proliferation assays and invasion assays were used to verify the effect of active ingredients on the proliferation and invasion of glioma cells.

**Results:**

A total of 73 potential targets of YQQYJDF in the treatment of GBM were obtained. Enrichment analyses showed that the biological processes and molecular functions involved in these target genes were related to the activation of the G protein-coupled receptor (GPCR) signaling pathway and the regulation of hypoxia. The neuroactive ligand‒receptor pathway, the cellular senescence pathway, the calcium signaling pathway, the cell cycle pathway and the p53 signaling pathway might play important roles. Combining the results of WGCNA and PPI analysis, five core target genes and their corresponding four core active ingredients were screened. Molecular docking indicated that the core active ingredient molecules and the proteins corresponding to the core target genes had strong binding affinities. Cell proliferation and invasion assays showed that the core active ingredients of YQQYJDF significantly inhibited the proliferation and invasion of glioma cells (*P* < 0.01).

**Conclusions:**

The present study predicted the possible active ingredients and targets of YQQYJDF in treating GBM, and analyzed its possible mechanism. These results may provide a basis and ideas for further research.

**Supplementary Information:**

The online version contains supplementary material available at 10.1186/s12906-023-04174-7.

## Background

Glioblastoma multiforme (GBM) is one of the most common human primary malignant brain tumors [1]. At present, the standard treatment for GBM is surgery combined with radiotherapy and chemotherapy, but the prognosis of GBM patients receiving standard treatment is still poor [1]. Therefore, it is very important to find more effective treatments to improve the prognosis of GBM patients, which is also a challenge for neurosurgeons.

Traditional Chinese medicine (TCM) has been used to treat brain tumors for thousands of years. The TCM classic “Zhongzang Jing”, which was written during the Han Dynasty in ancient China (around 200 AD), records the treatment of brain tumors with TCM. Many ingredients of TCM have been proven to have therapeutic effects on GBM [2, 3]. Yi Qi Qu Yu Jie Du Fang (YYQQJDF) is a TCM prescription for GBM based on classical TCM theory and data mining analysis of glioma cases of TCM [4, 5], which can improve the prognosis of patients with GBM in clinical application. This TCM prescription is composed of eight TCMs, including “Huangqi”, “Chuangxiong”, “Banxia”, “Baihuashecao”, “Gancao”, “Shancigu”, “Shichangpu” and “Taizishen”. However, due to the large number of active ingredients and targets of this TCM prescription, the mechanisms of its treatment effect in GBM are still unclear.

Network pharmacology is a method of understanding drug actions and interactions with multiple targets [6]. It uses bioinformatic methods to systematically catalog the molecular interactions of a drug. It is also an effective method of studying the mechanism of TCM prescriptions in treating diseases [7, 8]. The present study aimed to use a network pharmacology method to analyze the underlying mechanism of YQQYJDF in treating GBM.

## Methods

### GBM sample data collection

Transcriptome sequencing data and corresponding clinical information of The Cancer Genome Atlas (TCGA)-GBM cohort were obtained from the TCGA database [9] using the “TCGAbiolinks” package in R software (The R Foundation for Statistical Computing, Vienna, Austria). These data include 174 samples, of which 160 GBM samples have fully available survival data and five samples are normal tissue.

### Screening of Differentially Expressed Genes (DEGs)

Using the “limma” and “DESeq2” packages in R software, the differentially expressed mRNAs were identified between five normal and 169 tumor tissues in the TCGA-GBM cohort based on adjusted standards of *P* < 0.01 and | log_2_ (fold change) |> 2. The “ggplot” package was used to plot the volcano plot.

### Weighted Gene Coexpression Network Analysis (WGCNA)

The DEG data and clinical data of the TCGA-GBM cohort were used to construct the weighted gene coexpression network using the “WGCNA” package in R software to identify DEGs related to the overall survival (OS) of patients with GBM. The soft threshold power was seven.

### Selection of active ingredients of YQQYJDF

The formulas of YQQYJDF were “Huangqi” (*Hedysarum Multijugum Maxim.*), “Chuangxiong” (*Chuanxiong Rhizoma*), “Banxia” (*Arum Ternatum Thunb.*), “Baihuashecao” (*Hedyotis Diffusae Herba*), “Gancao” (*Liquorice*), “Shancigu” (*Pseudobulbus Cremastrae Seu Pleiones*), “Shichangpu” (*Acoritataninowii Rhizoma*) and “Taizishen” (*Pseudostellariae Radix*). The related ingredients were retrieved from the Traditional Chinese Medicine Systems Pharmacology Database and Analysis Platform (TCMSP) [10] and Traditional Chinese Medicine database [11]. Ingredients with oral bioavailability (OB) ≥ 30% and drug likeness (DL) ≥ 0.18 were selected as the active ingredients.

### Prediction of the targets of YQQYJDF active ingredients

The targets of the active ingredients of YQQYJDF were retrieved from the TCMSP database. The gene symbols and Entrez ID of targets were obtained by the “org.Hs.eg.db” package in R software.

### Prediction of potential targets of YQQYJDF in the treatment of GBM

The target genes of YQQYJDF active ingredients and DEGs of GBM were matched using a Venn diagram with Venny 2.1 [12], and the intersecting genes were obtained as potential target genes for YQQYJDF in the treatment of GBM.

### Enrichment analysis

The “clusterProfiler”, “org.Hs.eg.db”, “enrichplot” and “pathview” packages in R software with *p* value cutoff = 0.05 and q value cutoff = 0.05 were used to perform Gene Ontology (GO) enrichment analysis and Kyoto Encyclopedia of Genes and Genomes (KEGG) [13] signal pathway analysis for these target genes. The “ggplot2” package was used to visualize the results. The network graph of the relationship between target genes and pathways was drawn using Cytoscape 3.9.1 software (Cytoscape Consortium, USA).

### Protein‒protein interaction (PPI) analysis

The STRING database [14] was used to perform a PPI analysis to reveal the interaction network relationship of target genes at the translation level. The network graph of PPI was drawn by Cytoscape 3.9.1 software (Cytoscape Consortium, USA).

### Prediction of the core target genes of YQQYJDF for GBM treatment

The core target genes of YQQYJDF for the treatment of GBM were obtained by using the Venn diagram to intersect genes closely related to the patient’s prognosis obtained from WGCNA with the target genes of the active ingredients of YQQYJDF, the DEGs of GBM and the genes with a degree value greater than 10 in the PPI network.

### Molecular docking

The core active ingredients of YQQYJDF were obtained by matching the core target genes of YQQYJDF for GBM treatment and the target genes of YQQYJDF active ingredients. The 2D structure of the active ingredient small molecule ligand was obtained from the Drugbank database [15]. The 3D structures of the proteins of the core target genes were obtained from the RCSB protein data bank database [16]. Molecular docking was performed with AutoDockTools software 1.5.7 (Molecular Graphics Laboratory, The Scripps Research Institute). It is generally believed that the smaller the binding energy, the more stable the docking modules. A binding energy value less than -5.0 kcal/mol indicates that there is a good affinity between the receptor and the ligand, and a value below -7.0 kcal/mol indicates that there is a very strong affinity between the receptor and the ligand. Visualization of molecular docking results was performed with PyMOL software 2.5.4 (Schrödinger, LLC.).

### Cell culture and preparation of drugs

The U87MG human glioblastoma cell line was purchased from the Cell Resource Center of the Chinese Academy of Sciences (Shanghai, China). U87 cells were cultured in Dulbecco’s modified Eagle’s medium (Gibco; Thermo Fisher Scientific,Inc. USA) with 10% fetal bovine serum (FBS) (Gibco; Thermo Fisher Scientific,Inc. USA) at 37 °C in the presence of 5% CO_2_. Luteolin, quercetin and kaempferol were purchased from Sigma‒Aldrich (St. Louis, USA). Stigmasterol was purchased from Avanti Polar Lipids, Inc. (Alabaster, USA). All drugs were dissolved in dimethyl sulfoxide (DMSO) and stored at -20 °C.

### Cell proliferation assay

The Cell Counting Kit-8 (CCK-8) (Elabscience, China) assay was used to analyze the proliferation of glioma cells. U87 cells were seeded at a density of 5 × 10^3^ cells/well in 24-well plates. The cells were incubated in medium containing 40 μmol/L luteolin, quercetin, kaempferol or stigmasterol. The control group was treated with an equal volume of the solvent (DMSO) in the culture medium. After 24 h, glioma cells were then analyzed by CCK-8 assay according to the manufacturer’s instructions. Briefly, 50 μL CCK-8 solution was added to each well. After incubating at 37 °C for 4 h, the absorbance value at 450 nm was measured.

### Matrigel invasion assay

U87 cells were seeded at a density of 5 × 10^4^ cells/well in Transwell chambers (BD Biosciences, Bedford, USA) precoated with 50 μl Matrigel (BD Biosciences) diluted with culture medium. Serum-free culture medium containing 40 μmol/l luteolin, quercetin, kaempferol or stigmasterol was used to incubate the U87 cells and medium containing 20% FBS was used in the lower chamber as the chemoattractant. After 24 h, noninvasive cells were removed with cotton swabs, and the invasive cells were dyed with 0.1% crystal violet (Sigma‒Aldrich) and analyzed directly under an inverted fluorescent microscope (magnification, × 200, IX51; Olympus Corporation, Japan). The average cell number in five random visual fields was considered to represent the number of invasive cells of each group.

### Statistical analysis

Values are presented as the mean ± standard deviation. Data were analyzed using SPSS 25.0 software (SPSS, Inc. USA). One-way analysis of variance was used to compare the groups, and the least significant difference post hoc test was performed to further assess intergroup differences. A *P* value < 0.05 indicated a statistically significant difference.

## Results

### DEGs between GBM tissues and normal tissues

**Fig. 1:**
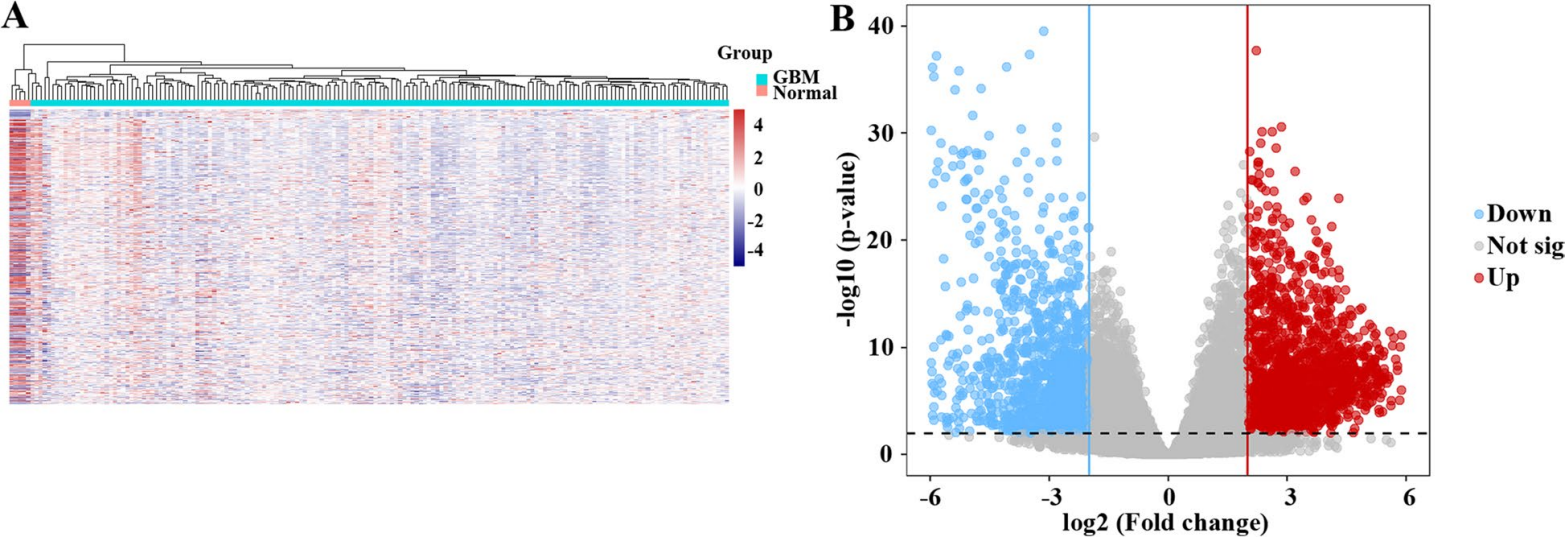
Differentially expressed genes between GBM tissues and normal tissues. **A** Heatmap; **B** Volcano map

As shown in Fig. 1, 3100 DEGs were identified between normal tissues and GBM tissues from the TCGA-GBM cohort; 1415 of the DEGs were upregulated and 1685 of the DEGs were downregulated in GBM.

### Identification of GBM clinical module genes by WGCNA

**Fig. 2:**
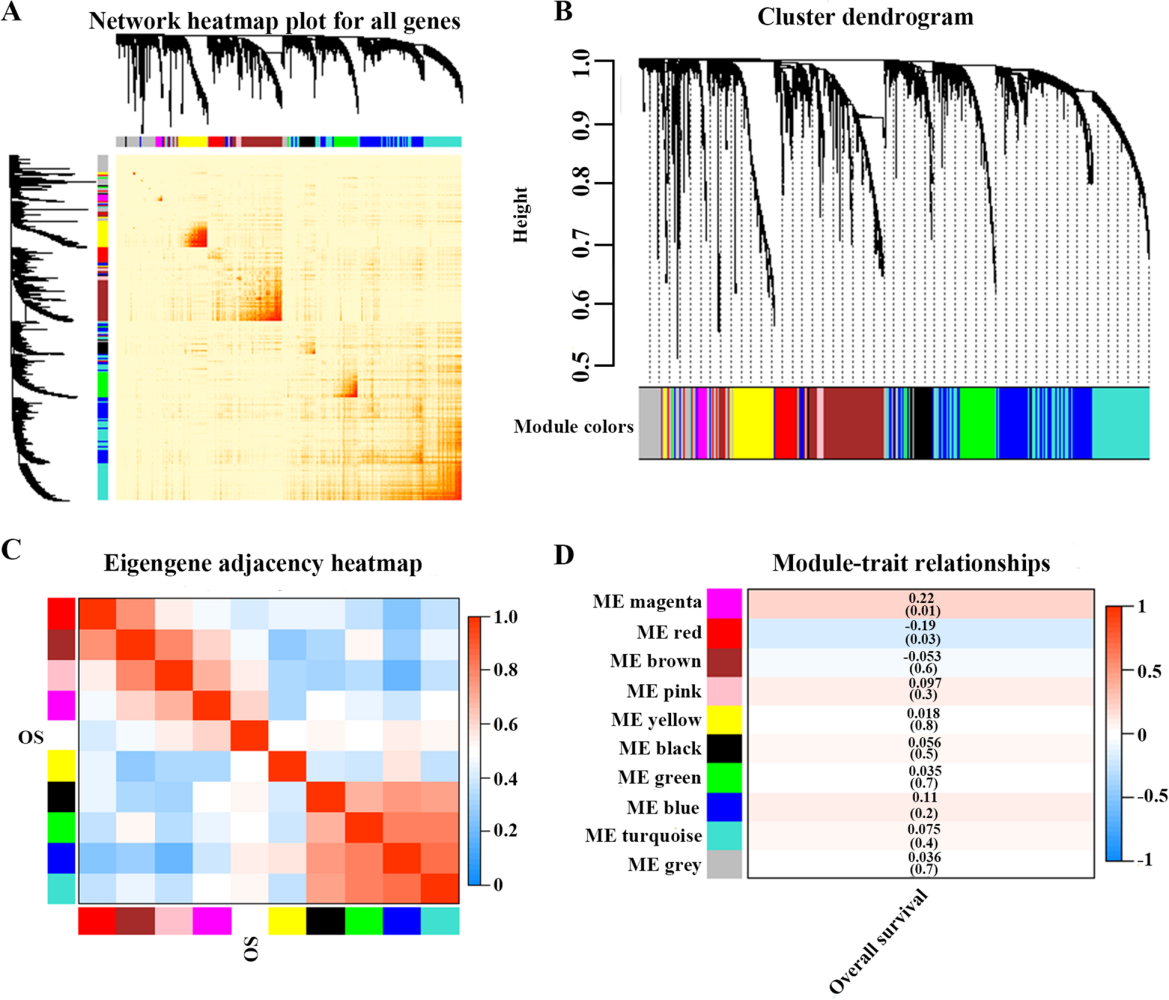
Weighted gene coexpression network analysis (WGCNA) of differentially expressed genes in GBM. **A** Network heatmap plot; **B** Cluster dendrogram; **C** Eigengene adjacency heatmap; **D** Module-trait relationships

Expression data for 3100 DEGs and the OS data of GBM patients were used to construct a scale-free network by WGCNA. As shown in Fig. 2, nine modules were identified; including black, blue, magenta, red, yellow, brown, green, gray, pink and turquoise. According to the results of the module-trait relationship analysis, a total of 236 genes in the magenta and red modules (shown in Table 1 and Supplementary Table 1) were considered to be closely related to the prognosis of GBM patients (*P* < 0.05).

**Table 1:** WGCNA color modules related to the prognosis of GBM patients

Module	Number of genes	*P* value
Magenta	59	0.01
Red	176	0.03
Sum	235	

### Active ingredients and potential target genes of YQQYJDF

After screening, a total of 119 active ingredients and 252 corresponding target genes of YQQYJDF were obtained (Supplementary Table 2).

### Prediction of YQQYJDF targets in the treatment of GBM

**Fig. 3:**
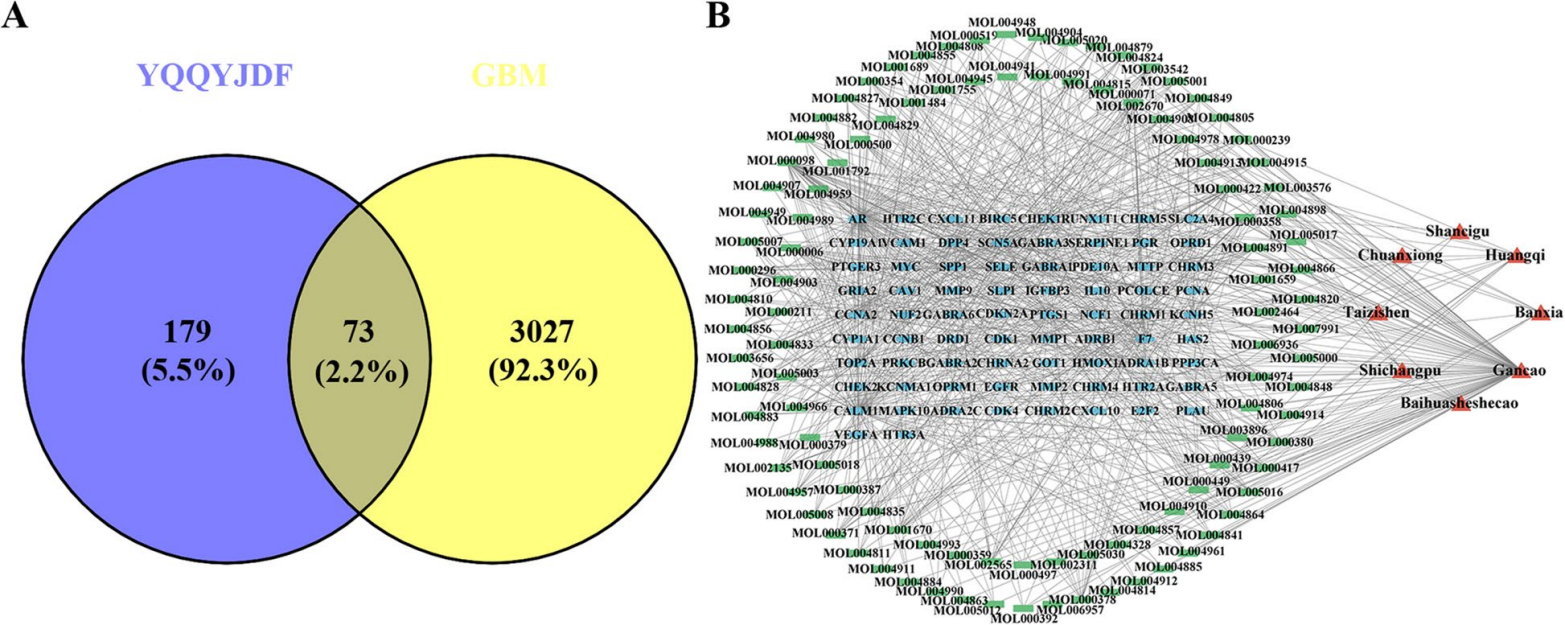
YQQYJDF targets in the treatment of GBM. **A**Venn diagram of potential target genes of YQQYJDF and DEGs in GBM; **B** The TCM-active ingredient-target network. Red nodes: TCMs, green nodes: active ingredients, blue nodes: targets

As shown in Fig. 3 and Supplementary Table 3, after taking the intersection of YQQYJDF potential target genes and DEGs of GBM, a total of 73 YQQYJDF potential targets in the treatment of GBM were obtained.

### Enrichment analysis of potential targets of YQQYJDF in the treatment of GBM

**Fig. 4:**
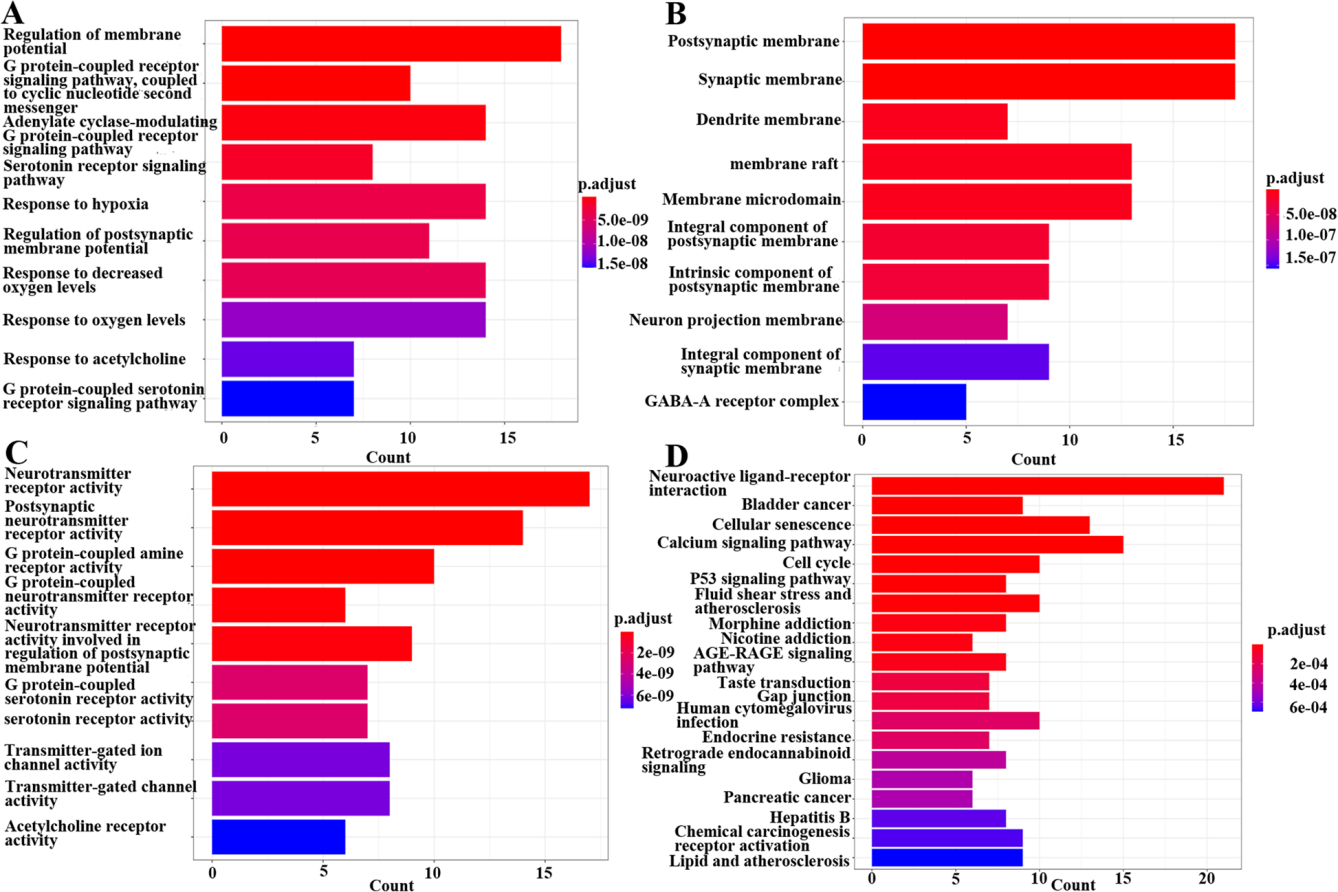
Enrichment analysis of potential targets of YQQYJDF. **A**, **B** and **C** The top 10 enriched terms from the GO analysis. **A** Biological process (BP) terms; **B** cellular component (CC) terms; **C** molecular function (MF) terms. **D** The top 20 pathways of KEGG analysis

A total of 807 GO items were obtained by GO enrichment analysis of 73 potential YQQYJDF targets in the treatment of GBM; There were 656 enriched biological process (BP) terms, 57 enriched cellular component (CC) terms and 94 enriched molecular function (MF) terms. Figure 4A, B and C show the top 10 enriched terms in each of the three categories. The length of the line in the figures represents the number of genes enriched in this term, and the color represents the significance of the enrichment. The results showed that the mechanism of YQQYJDF in the treatment of GBM might be related to several biological processes such as regulation of membrane potential, the G protein-coupled receptor signaling pathway, the serotonin receptor signaling pathway, the response to hypoxia, and leukocyte migration. Related molecular functions included neurotransmitter receptor activity, G protein-coupled receptor activity, serotonin receptor activity, etc. Moreover, some cellular components, such as postsynaptic membrane, synaptic membrane, dendrite membrane and membrane raft, were involved.

Sixty-eight pathways were obtained through KEGG pathway enrichment analysis. The top 20 pathways are shown in Fig. 4D. The length of the line represents the number of genes enriched in the pathway; the color represents the significance of the enrichment. The results showed that the potential targets of YQQYJDF in the treatment of GBM were related to the pathways that regulate neuroactive ligand‒receptor interactions, malignant tumors, the cell cycle, and cellular senescence.

### PPI network analysis

**Fig. 5:**
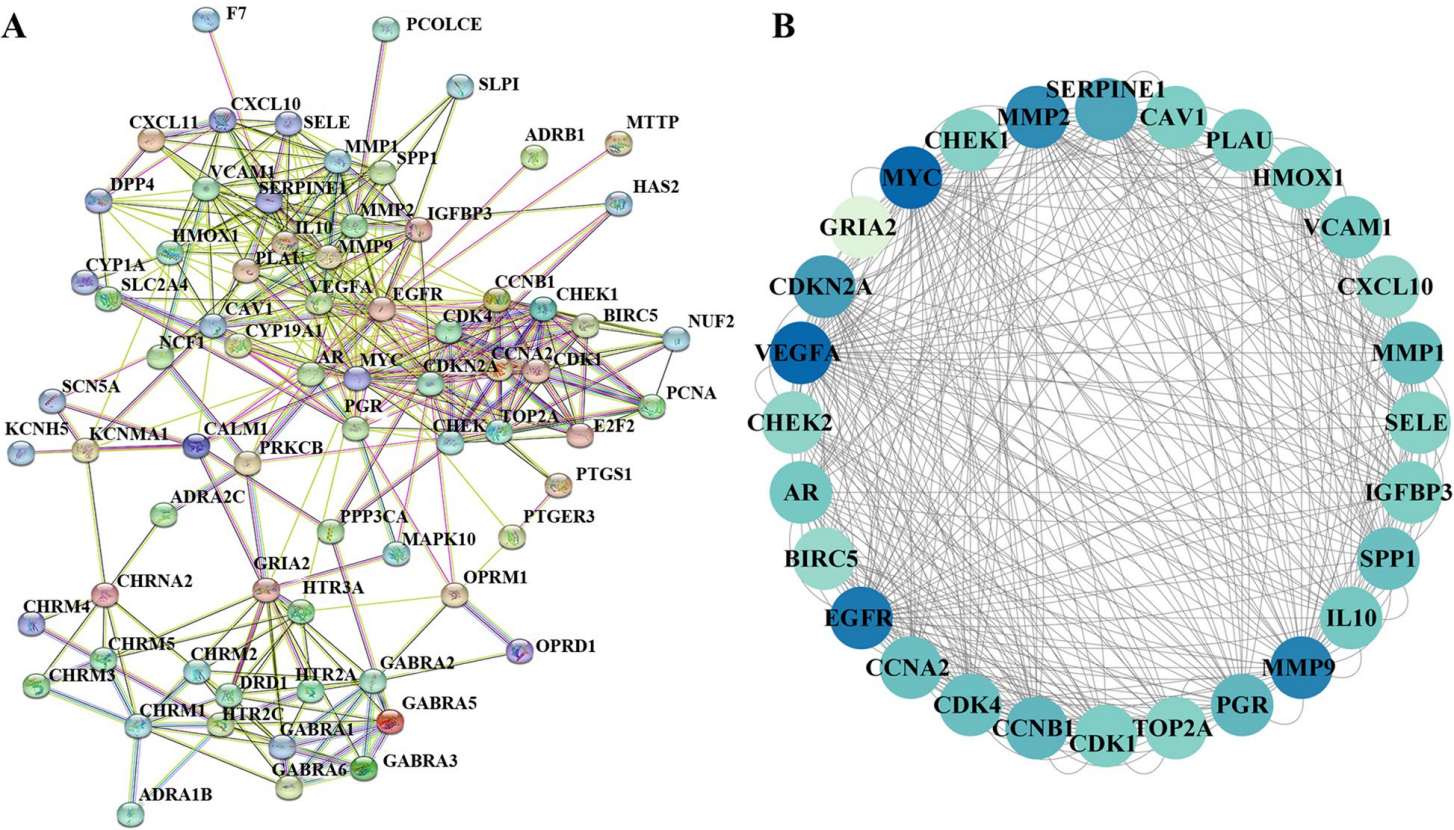
The interaction of the potential target genes of YQQYJDF in the protein‒protein interaction network. **A** PPI network, **B** The 28 genes with a PPI network degree greater than 10

As shown in Fig. 5A, the PPI network showed the interaction of potential target genes at the protein level. Figure 5B shows the 28 genes with a PPI network degree greater than 10. The darker the color is, the higher the degree value, indicating that the corresponding protein is more important in the network.

### Selection of the core target genes of YQQYJDF for GBM treatment

**Fig. 6:**
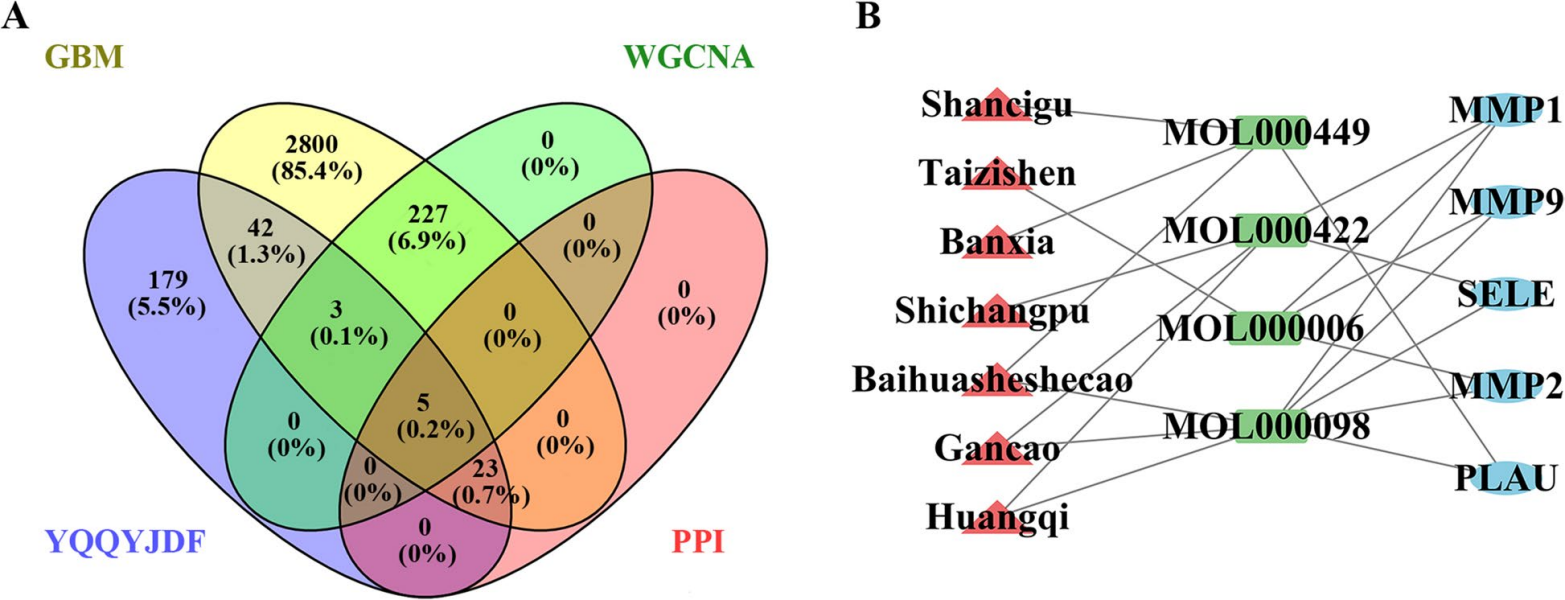
The core target genes of YQQYJDF. **A** Venn diagram of potential target genes of YQQYJDF, DEGs of GBM, WGCNA screening genes and genes with a PPI network degree greater than 10. **B** The network of YQQYJDF core targets

As shown in Fig. 6, after taking the intersection of YQQYJDF potential target genes, WGCNA screening genes, genes with a degree value greater than 10 in the PPI network and DEGs of GBM, five core target genes of YQQYJDF that are very important in the PPI network and closely related to the prognosis of glioma patients were obtained (Table 2).

**Table 2:** The core target genes of YQQYJDF for GBM treatment

Gene symbol	Gene name
MMP1	Interstitial collagenase
MMP2	Matrix metalloproteinase-2
MMP9	Matrix metalloproteinase-9
PLAU	Urokinase-type plasminogen activator
SELE	E-selectin

### Molecular docking results

**Fig. 7:**
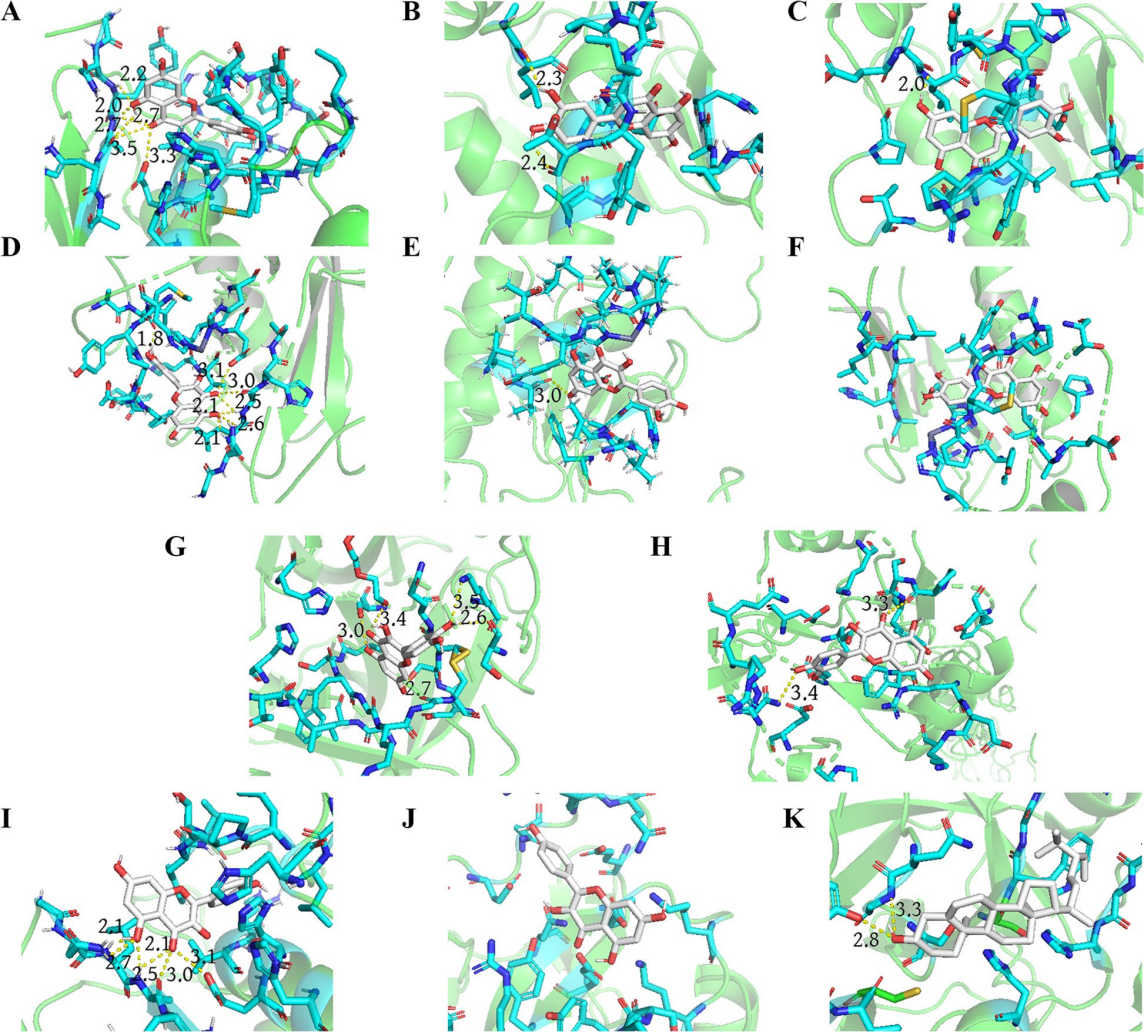
Molecular docking of proteins expressed by core target genes with the corresponding active ingredients of YQQYJDF. **A** Luteolin with MMP-1, **B** Luteolin with MMP-2, **C** Luteolin with MMP-9, **D** Quercetin with MMP-1, **E** Quercetin with MMP-2, **F** Quercetin with MMP-9, **G** Quercetin with PLAU, **H** Quercetin with SELE, **I** Kaempferol with MMP-1, **J** Kaempferol with SELE, **K** Stigmasterol with PLAU

Molecular docking of proteins encoded by five core target genes with the corresponding core active ingredients showed that their binding energies were all less than -5.0 kcal/mol, indicating that there are good binding effects between the target proteins and active ingredients (Table 3). The visualized results of molecular docking are shown in Fig. 7.

**Table 3:** The molecular docking results

Target	Active ingredient	Molecule ID	Binding energy (kcal/mol)
MMP1	Luteolin	MOL000006	-10.1
MMP2	Luteolin	MOL000006	-7.8
MMP9	Luteolin	MOL000006	-10.0
MMP1	Quercetin	MOL000098	-10.1
MMP2	Quercetin	MOL000098	-7.0
MMP9	Quercetin	MOL000098	-10.0
PLAU	Quercetin	MOL000098	-8.0
SELE	Quercetin	MOL000098	-5.3
MMP1	Kaempferol	MOL000422	-9.7
SELE	Kaempferol	MOL000422	-5.1
PLAU	Stigmasterol	MOL000449	-6.1

*MMP1* Matrix metalloproteinase-1/ Interstitial collagenase, *MMP2* Matrix metalloproteinase-2, *MMP9* Matrix metalloproteinase-9, *PLAU* Urokinase-type plasminogen activator, *SELE* E-selectin

### The core active ingredients of YYQQJDF inhibit the proliferation of glioma cells

**Fig. 8:**
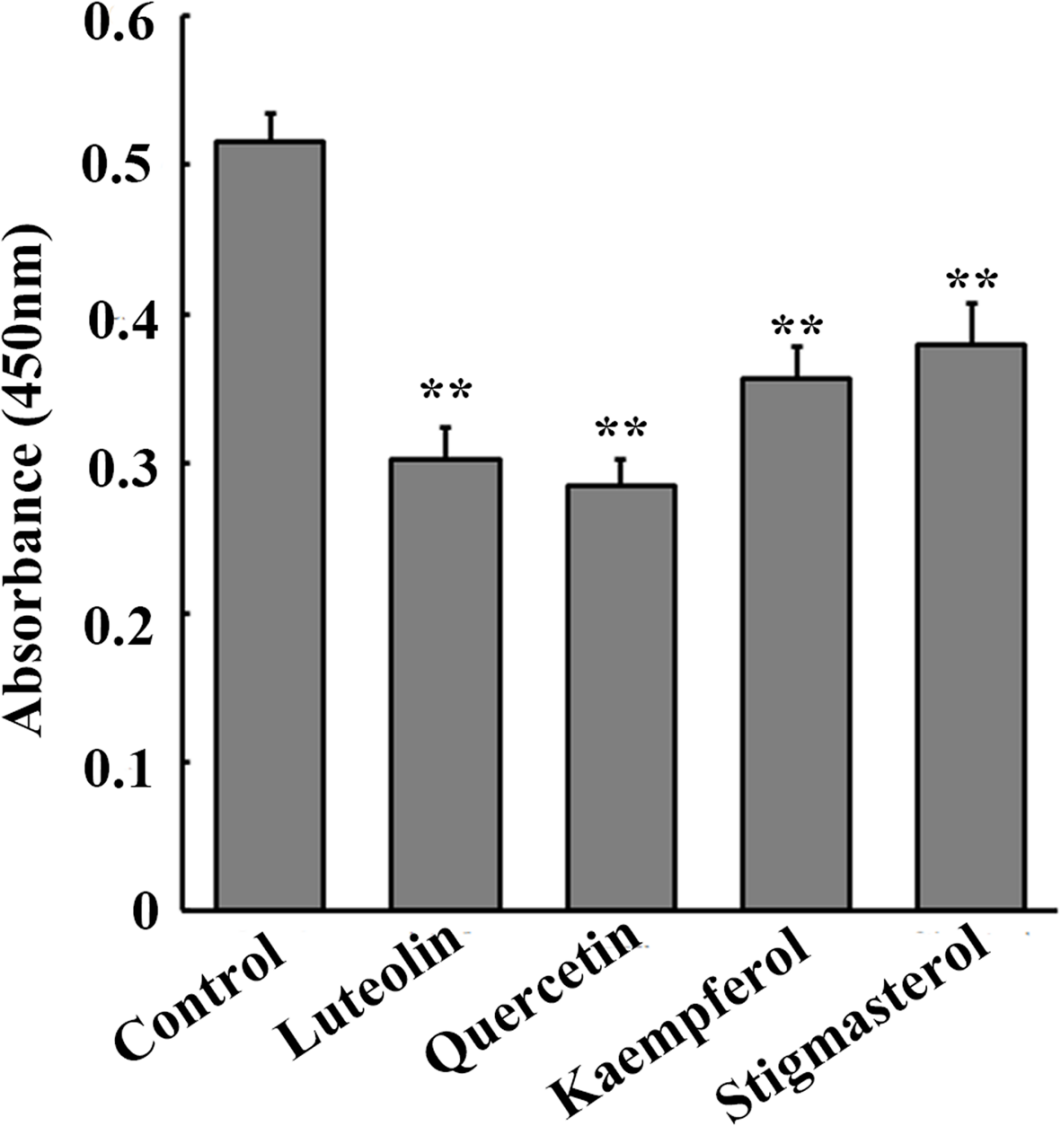
The effect of YQQYJDF core active ingredients on proliferation of U87 glioma cells. **, *P* < 0.01

After treatment with the core active ingredients of YYQQJDF, the proliferation ability of U87 glioma cells was detected by CCK-8 assay. As shown in Fig. 8, after 24 h of intervention, four core active ingredients luteolin, quercetin, kaempferol and stigmasterol significantly inhibited the proliferation of U87 glioma cells (*F* = 52.075, *P* < 0.01).

### The core active ingredients of YYQQJDF inhibit the invasion of glioma cells

**Fig. 9:**
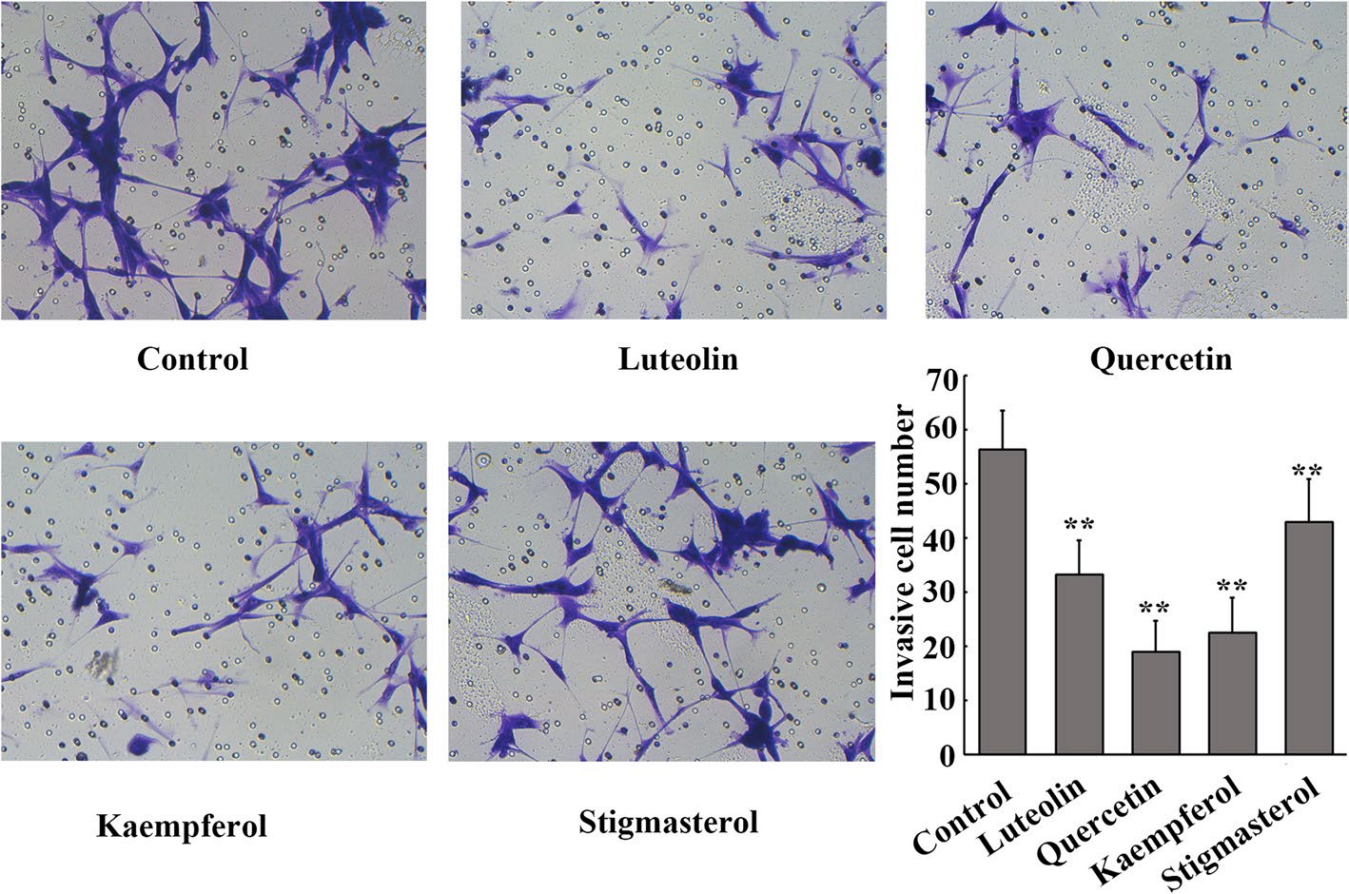
The effect of YQQYJDF core active ingredients on invasion of U87 glioma cells. **, *P* < 0.01

Following treatment with the four core active ingredients of YYQQJDF for 24 h, the number of invading glioma cells was significantly reduced compared with that in the control group (*F* = 25.648, *P* < 0.01) (Fig. 9).

## Discussion

Although TCM has been used to treat various diseases for thousands of years, it is difficult to gain recognition in the field of modern medicine due to theoretical differences and the lack of direct experimental evidence [17]. For example, the principles of TCM such as the concept of “vital energy”, are esoteric and difficult to validate under modern medicine methods. The diagnostic and treatment methods of TCM are also fundamentally different from modern medicine [18]. Therefore, research on the active ingredients and biological functions of TCM has always been the focus of TCM pharmacological research [19]. The results of these studies are helpful to reveal the mechanism of TCM in treating diseases [20, 21]. Recently, network pharmacological analysis has been increasingly applied to TCM research. Network pharmacology can be used to search for the relationships among herbs, active ingredients, diseases and target genes, predict key active ingredients, targets and possible mechanisms of TCM in the treatment of diseases, and provide clues for further research [7, 8]. In the present study, network pharmacological methods were used to analyze the potential mechanisms of YQQYJDF in treating GBM.

According to the results, five core target genes and four corresponding active ingredients were obtained. Among the five core target genes, MMP-1 is considered closely related to the malignant degree of glioma [22] and the prognosis of glioma patients [23]. Similar to other members of the MMP family, MMP-1 can not only target the extracellular matrix, but also activate other bioactive molecules and activate downstream proinvasive and prooncogenic signaling mechanisms [24]. Inhibition of MMP1 expression can significantly inhibit glioma proliferation and invasion [25]. MMP-2 and MMP-9 are often discussed together due to their similar mechanisms and signaling pathways. As two key members of the MMP family, they are closely related to the migration, proliferation, vascular mimicry formation, and tumor growth of glioma [26, 27]. Many interventions can inhibit glioma growth by inhibiting the expression of MMP-2 and MMP-9 [28, 29]. PLAU participates in a series of cell physiological activities, such as migration and invasion [30]. Abnormal expression of PLAU is associated with the development of glioma and the prognosis of glioma patients[31]. Upregulation of PLAU also promotes the migration of glioma cells [32]. SELE is a selectin cell adhesion molecule expressed on endothelial cells induced by cytokines, that plays an important role in inflammation [33]. At present, SELE is considered to be related to the progression, metastasis and development of various tumors [34, 35]. Few studies have examined the relationship between SELE and glioma. A study found that SELE can promote glioma metastasis [36].

In YQQYJDF, the core active ingredients corresponding to these five target genes are luteolin, quercetin, kaempferol and stigmasterol. Luteolin is a natural flavone compound that is considered to have antitumor effects [37, 38]. Some studies have confirmed that luteolin can significantly inhibit glioma cell proliferation, migration, and invasion and induce their apoptosis [39, 40]. These effects may be related to the activation of the MAPK pathway [39] or disruption of the function of RNA binding proteins [40]. Quercetin is another recognized small molecule compound with antitumor effects [41, 42]. This natural compound can affect a variety of cellular and molecular processes of tumors, such as apoptosis, metastasis, and autophagy [43]. The antiglioma effect of quercetin involves multiple mechanisms, including apoptosis induction, metastasis and invasion inhibition, and cytotoxicity induction [44–46]. Kaempferol has also shown antitumor effects in many studies[47]. Studies have shown that kaempferol can inhibit the proliferation, metabolism and migration of glioma cells in vitro and inhibit tumor growth in vivo [48, 49]. These effects may be related to programmed cell death and oxidative stress [48, 50]. Different from the other three active ingredients, stigmasterol is a sterol compound, that has the main function of maintaining the structure and physiology of cell membranes [51]. There are few studies on the effect of stigmasterol on glioma. In one study, researchers found that stigmasterol had cytotoxic effects on glioma cells [52]. The results of the present study verified that these four core active ingredients can significantly inhibit the proliferation and invasion of glioma cells. These core active ingredients may play an important role in the antiglioma effects of YDDYJDF.

In addition to the five core target genes, 68 other potential target genes of YQQYJDF for GBM treatment were obtained in this study. According to the results of the GO analysis, many of the biological processes and molecular functions involving in these target genes are related to the activation of the G protein-coupled receptor (GPCR) signaling pathway and the regulation of hypoxia. GPCRs and their downstream signaling targets play a central role in the initiation and progression of tumors [53]. They can regulate the growth and survival of tumor cells, and provide nutrients and routes for tumor metastasis by inducing cytoskeletal changes and angiogenesis [54]. GPCR signaling pathways are also important for the tumorigenesis and development of glioma [55]. Therefore, they are also considered potential targets for the treatment of glioma [56]. Hypoxia can promote glioma growth, angiogenesis, and invasion, which is achieved mainly through the hypoxia-inducible factor 1 (HIF-1) pathway to upregulate target genes such as vascular endothelial growth factors (VEGF), VEGF receptors (VEGFR), and MMPs [57]. The mechanism of YQQYJDF in the treatment of GBM may be related to regulating the biological processes involved in the GPCR signaling pathway and hypoxia. In the KEGG analysis, the neuroactive ligand‒receptor pathway, cellular senescence pathway, calcium signaling pathway, cell cycle pathway, p53 signaling pathway and other signaling pathways were considered to be related to YQQYJDF treatment of GBM. As a classical tumor-related signaling pathway, the p53 signaling pathway has long been a focus of research. Many interventions have been proven to regulate the biological behavior of glioma from different aspects through the p53 signaling pathway [58, 59]. The cell cycle pathway participates in cell cycle regulation as a downstream pathway of many signaling pathways [60, 61]. In glioma, interventions can regulate the cell cycle pathway through Akt, PI3K and other signaling pathways to regulate the glioma cell cycle and apoptosis [62, 63]. The calcium signaling pathway is also crucial for the behavior of glioma cells [64]. The calcium signal may regulate glioma cell motility, which is important in the early stages of tumor development [64]. Similar to the cell cycle pathway, cellular senescence is regulated by upstream pathways [65]. Many interventions can inhibit glioma cell proliferation through the cellular senescence pathway [66, 67]. The neuroactive ligand‒receptor pathway involves various neuroactive substances, such as acetylcholine, dopamine, and 5-hydroxytryptamine (5-HT) [68]. Acetylcholine participates in the regulation of astrocyte differentiation into glioma cells [69]. Dopamine can induce glioma cell apoptosis and inhibit tumor growth by regulating mitochondrial apoptosis and anti-inflammatory signaling pathways [70]. 5-HT can induce the expression of glial cell line-derived neurotrophic factor (GDNF) in glioma cells [71], which is an important prerequisite for the initiation and development of glioma [72]. However, the above biological processes and signaling pathways involved in the possible mechanism of YQQYJDF treatment of GBM need to be further verified in vivo and in vitro. In addition, according to the GO analysis and KEGG results, the mechanism of YQQYJDF in treating GBM may involve other biological processes and signaling pathways, but this needs to be verified in further studies. These results may provide potential new research directions.

There are still some limitations to this study. First, the ingredients and target genes in this study are mainly from databases. Due to the limitations of data sources and differences in screening strategies, some ingredients or target genes may be omitted. Second, this is a study based on network pharmacology and molecular docking, and the results have been preliminarily verified in vitro. Further in vitro and in vivo experiments are needed to verify these possible mechanisms. These shortcomings will be addressed in further research.

## Conclusions

In conclusion, the present study involved the prediction of the possible active ingredients and targets of YQQYJDF in the treatment of GBM using network pharmacology and the analysis of its possible mechanism. These results may provide a basis and ideas for further research.

### Supplementary Information

**Additional file 1: Supplementary Table 1.** Genes in WGCNA color modules related to the prognosis of GBM patients.

**Additional file 2: Supplementary Table 2.** Active ingredients and target genes of YQQYJDF.

**Additional file 3: Supplementary Table 3.** YQQYJDF targets in the treatment of GBM.

## Data Availability

The data in the current study come from TCGA database (https://www.cancer.gov/about-nci/organization/ccg/research/structural-genomics/tcga), TCMSP database (https://old.tcmsp-e.com/tcmsp.php), TCM database (http://tcm.cmu.edu.tw/), STRING database (https://string-db.org/), Drugbank database (https://go.drugbank.com/) and RCSB PDB database (https://www.rcsb.org/). The data supporting the conclusion of the study can be obtained from the corresponding author upon request.

## Abbreviations

GBM: Glioblastoma multiforme
TCM: Traditional Chinese medicine
YQQJDF: Yi Qi Qu Yu Jie Du Fang
TCGA: The cancer genome atlas
DEG: Differentially expressed genes
WGCNA: Weighted gene coexpression network analysis
STRING: Search Tool for the Retrieval of Interacting Genes/Proteins
OS: Overall survival
TCMSP: Traditional Chinese Medicine Systems Pharmacology Database and Analysis Platform
OB: Oral bioavailability
DL: Drug likeness
GO: Gene ontology
KEGG: Kyoto encyclopedia of genes and genomes
PPI: Protein-protein interaction
DMSO: Dimethyl sulfoxide
CCK-8: Cell counting kit-8
FBS: Fetal bovine serum
MMP1: Matrix metalloproteinase-1/Interstitial collagenase
MMP2: Matrix metalloproteinase-2
MMP9: Matrix metalloproteinase-9
PLAU: Urokinase-type plasminogen activator
SELE: E-selectin
5-HT: 5-Hydroxytryptamine
GPCR: G protein-coupled receptor
HIF-1: Hypoxia-inducible factor 1
VEGF: Vascular endothelial growth factor
VEGFR: Vascular endothelial growth factor receptor
